# Acceptability of a technology-supported and solution-focused intervention (DIALOG+) for chronic depression: views of service users and clinicians

**DOI:** 10.1186/s12888-021-03256-5

**Published:** 2021-05-20

**Authors:** Aleksandra Matanov, Philip McNamee, Syeda Akther, Nick Barber, Victoria Bird

**Affiliations:** 1 The Unit for Social and Community Psychiatry, WHO Collaborating Centre for Mental Health Services Development, Queen Mary University of London, NCfMH, London, E13 8SP UK; 2 East London NHS Foundation Trust, NCfMH, London, E13 8SP UK

**Keywords:** DIALOG+, Chronic depression, Digital intervention, Solution-focused, Community mental health

## Abstract

**Background:**

Treating chronic depression represents a significant burden for the NHS, yet there is a lack of evidence-based interventions and research specifically focused on this condition. DIALOG+, a technology-assisted and resource-oriented intervention found effective for people with psychosis, may improve care for this service user group. The aim of this study was to explore the acceptability and relevance of DIALOG+ for the treatment of chronic depression in community-based settings.

**Methods:**

A convenience sample of 16 mental health professionals and 29 service users with chronic depression tested the DIALOG+ intervention in routine community care appointments for 3months across 3 different mental health NHS Trusts in England. Of these, 15 clinicians and 19 service users were individually interviewed about their experiences. Interview transcripts were analysed using thematic analysis by an analytic team which included a service user researcher.

**Results:**

Analysis of the combined dataset identified five overarching themes: DIALOG+ Structure; Therapeutic Communication; Reflecting and Monitoring; Empowerment and Powerlessness; and The Impact of Technology. Overall, service users and clinicians were interested in the continued use of DIALOG+ as part of routine care.

**Conclusions:**

DIALOG+ was viewed as acceptable by both service users with chronic depression and their clinicians who work in community care settings, albeit with some caveats. Clinician training required significant improvements to address the issues that were referenced, most notably around support with using technology.

**Supplementary Information:**

The online version contains supplementary material available at 10.1186/s12888-021-03256-5.

## Background

Depression is a leading cause of disability worldwide [1] and a priority within the National Health Service (NHS) [2]. The number of people experiencing depression within the UK is set to increase to 1.45 million by 2026 [3]. Despite the availability of a range of treatment strategies for acute episodes of depression, up to 30% of people do not adequately improve and instead go on to develop a chronic disorder [4]. Chronic depression is associated with poor clinical and social outcomes including an increased suicide risk, poor quality of life, physical comorbidity, reduced social networks, functional impairment as well as high economic costs [3,5]. Past research has tended to focus on the treatment of acute depression, resulting in a lack of evidence-based interventions specifically tailored for chronic forms [6]. Where treatment options do exist, they lack effectiveness, or require referrals to specialist services [7]. Consequently, there is a need to develop interventions that are both clinically and cost-effective and can be routinely implemented within different clinical settings.

DIALOG+, an app-based and resource-oriented intervention, represents one possible solution. This intervention structures communication between service users and their clinicians during routine meetings in mental health care settings, aiming to create better treatment plans and improve outcomes. It draws on concepts from quality of life research, patient-centred communication, IT developments and solution-focused therapy. DIALOG+ consists of a patient-centred assessment whereby service users rate their satisfaction with different areas of life and treatment, on a tablet computer. Several areas are then selected for more detailed discussions guided by a four-step approach which is informed by the principles of brief solution-focused therapy.

The intervention was initially developed to make routine meetings in community care more clinically effective for people with psychosis [8, 9]. A cluster randomised controlled trial with this population found that those who used the intervention once a month for 6months had better quality of life, fewer unmet needs, lower general symptom levels, better social outcomes and lower NHS treatment costs [9].

The aim of the Tackling Chronic Depression (TACK) Programme (RP-PG-0615-20010) is to adapt DIALOG+ to the needs of service users with chronic depression and test its clinical and cost effectiveness. In order to investigate the acceptability of the intervention for this patient group, and if any amendments to the intervention were required prior to trial work, we conducted an exploratory qualitative study. Acceptability was defined broadly as perceived advantages and disadvantages of the intervention, in addition to a specific survey question about intentions for continued use beyond the study. Mental health professionals, and service users from their caseloads, were invited to try the DIALOG+ during their routine meetings for 3months in order to gain practical knowledge of how to use it. They were interviewed about their experiences after the end of the intervention delivery period and their responses analysed using thematic analysis. The findings of our exploratory work are presented in this paper.

## Methods

### Design, setting and participants

The present study had an exploratory, qualitative design focused on eliciting views from service users who have chronic depression and mental health professionals who treat them in community-based settings. It was carried out in the initial, exploratory phase of the TACK Programme in order to explore the acceptability and relevance of the DIALOG+ intervention for this service user population.

We recruited mental health professionals from community-based services and service users from their caseloads to gain experience of using the DIALOG+ intervention for 3months during routine meetings scheduled within that period. Participants were asked to use the intervention at least once per month with an option to use it more, if so inclined. The participants agreed to be interviewed about their experiences in post-intervention qualitative assessments conducted on a one-to-one basis.

The participating clinicians and service users were recruited from three NHS England mental health Trusts: East London NHS Foundation Trust, Oxford Health NHS Foundation Trust and Gloucestershire Health and Care NHS Foundation Trust. The TACK researchers presented the study to a variety of clinicians with different professional backgrounds working across community-based services. Following their recruitment, the clinicians screened their caseloads and approached potentially eligible service users, providing them with information about the study. Interested service users were contacted by a researcher who explained the study in greater detail, answered any queries and obtained informed consent.

Service users were eligible for participation if they had exhibited symptoms of depression (consistent with the ICD-10 diagnoses F3234 [depressive episode (F32), recurrent depression (F33) or persistent mood disorder (F34)]) for at least 2 years [10]. They were identified and assessed for eligibility by their treating clinicians who based these assessments on their knowledge of and familiarity with service users case histories and current clinical presentation. The minimum duration was set to 2 years based on the presence of this criterion in the classification systems and the National Institute for Health and Care Excellence (NICE) clinical guidance, as well as frequent use in the research literature [10,13]. As depression is known to be highly co-morbid with other psychiatric disorders it was acceptable for service users to have more than one psychiatric diagnosis [14]. The full eligibility criteria for both mental health professionals and service users are listed in Table1.

**Table 1:** Participants eligibility criteria

Service users	Mental health professionals
1870years old	Qualification as a mental health professional
Clinical diagnosis of depression with a duration of illness of at least 2years	Experience of working in health care for at least 6 months
Using NHS mental health services for the treatment of depression	Currently (or within the last 6 months) treating individuals with chronic depression
Capacity to provide informed consent	No plans to leave their post within the next 3 months
Ability to speak and understand English	

Following service users recruitment, the participating clinicians were given training in the DIALOG+ intervention, and were asked to deliver at least one DIALOG+ session per month, within already occurring routine meetings. After 3months, all participants were invited to take part in a semi-structured interview about their experiences. Separate topic guides for service users and clinicians were created in collaboration with a service-user and carer advisory group. The topic guides covered the benefits and downsides of using DIALOG+, barriers to and facilitators of routine implementation, possible impact on therapeutic communication, app design and usability, and suggestions for improvement. There was also a specific closed question about intentions for future use. The clinician topic guide included questions related to the experience of receiving the DIALOG+ training.

The study was given a favourable opinion by the Wales Research Ethics Committee 6.

### DIALOG+ intervention

The DIALOG+ intervention is a novel technology-assisted intervention available as an app and delivered on a tablet computer within routine meetings in mental health care settings. The aim of DIALOG+ is to improve communication between service users and clinicians within their already occurring care meetings and to make these meetings more structured, patient-centred and recovery oriented. The ultimate objective is to create more personalised treatment plans for service users and improve outcomes. The intervention structures existing one-to-one appointments and does not require any additional sessions. Service users continue to receive any treatment as usual including medication and psychological therapies. The presence of the tablet allows service users to be more actively involved in the meetings, with the device easily passed between them and their clinician.

The DIALOG+ intervention was specifically developed as a generic and flexible tool to be used by a wide variety of mental health professionals, including those who act as care-coordinators. The delivery of the intervention can be adapted to the needs of individual clinicians and service users including personal therapeutic style and more practical considerations such as the number and frequency of sessions. The manual and the training were designed to be brief and accessible, with the latter lasting 6090min and delivered either one-to-one or in a group format.

Each DIALOG+ session begins with a patient-centred assessment during which service users rate their satisfaction with the 11 items of the DIALOG Scale: eight areas of life (mental health, physical health, job situation, accommodation, leisure activities, friendships, relationship with family/partner, personal safety) and three aspects of treatment (medication, practical help, meetings with professionals). Each item is rated from 1 (totally dissatisfied) to 7 (totally satisfied), followed by a question on whether the service user wants additional help with that area. The ratings are summarised on screen, allowing for comparisons with the ratings from previous meetings. Clinicians are instructed to review the ratings with service users and offer positive feedback on any improving or high-scoring areas. Based on this review, service users select up to three areas to discuss with their clinicians in more detail later on in the session. This discussion is guided by a four-step approach informed by the principles of brief solution-focused therapy. In this approach service users are encouraged to consider different strategies and resources they could use to address the concerns raised, with particular emphasis on what they can do themselves. The final step is to agree on actions to be completed before and then reviewed at the beginning of the next session. These items make up the more personalised care plan. The screenshots of the DIALOG+ app are included as supplementary files1, 2 and 3, and further description can be found in Priebe et al. [15].

The DIALOG+ intervention has been adopted for use in a variety of services across NHS England and NHS Wales, and is currently being tested globally in community care in over 20 countries.

### Data analysis

All interviews were audio recorded and transcribed verbatim, with any identifying information removed. The data was analysed using thematic analysis [16]. This method was chosen because it allows robust analysis yet makes it possible to present the findings in a way accessible to service users, health practitioners and other stakeholders outside of academia [17]. The study was conceptualised and analysis carried out within a realist paradigm to explore and report the participants experiences of receiving the DIALOG+ intervention.

The analysis was conducted by a four member analytic team (AM, PM, SA, NB). Two members were researchers with a professional background in psychology (AM & SA), while one was an academic psychologist with expertise in qualitative methods (PM). We felt it was important that individuals with relevant lived experience were able to contribute their views and perspectives during the analysis of collected data. Consequently, a researcher with personal experience of chronic depression was included in the team (NB). NB has some research experience from involvement activities in other studies but he also received a qualitative data analysis training course from the research team.

We broadly followed the 6-step approach outlined by Braun and Clarke [16] to analyse the data. In the initial step, AM read and re-read the transcripts to ensure familiarisation with the data, while writing notes and generating ideas for coding at the same time. An initial list of codes was created for the clinician data and service user data separately, and these were presented to and refined through the discussions with the analytic team. This resulted in two coding frames which were applied to the relevant transcripts. The coding was carried out by AM using NVivo software [18]. The progress of coding and the need for minor changes to the frames were regularly discussed and agreed in further meetings of the analytic team. The next stages of analysis involved an iterative process of searching for patterns in coded data, construction of broader categories and their subsequent refinement and grouping into conceptual themes. Although these steps were carried out separately for the clinician and service user data, the themes and subthemes developed from the two data sets were regularly compared and contrasted by the analytic team who observed that the clinicians and service users reported similar or complimentary views and experiences of using the DIALOG+ intervention. Consequently, the themes and categories from the two datasets were merged and both perspectives gradually combined into several overarching themes. We believe that integrating both perspectives added meaning and depth to the themes, and was a better way to utilise the data obtained from the two participant groups. A complete separation and discrete presentation of the two datasets would have led to repetitive and less meaningful findings in regard to our research objectives. The rudimentary themes were also periodically presented to and discussed with a wider service user and carer advisory team and the wider TACK study group, with their feedback integrated into the analysis.

During the process of analysing data and generating themes the members of the analytic team engaged in critical examination of the ways in which their contributions and interpretations were influenced by our professional backgrounds, education and personal experiences. The service user researcher NB contributed to this approach by his reading of and reflection on data rooted in his lived experience of chronic depression and using mental health services.

### Data protection

The investigators and researchers complied with the requirements of the Data Protection Act1998 [19] with regards to the collection, storage, processing and disclosure of personal information.

All participants were assigned a participant ID number used for data processing purposes and all data was pseudonymised to protect confidentiality. The individual interviews were audio-recorded with explicit permission of the participants and all the transcriptions completed by an NHS approved professional service. All identifiable information was removed from the resulting transcripts and replaced with pseudonymised labels.

Participant identifiable data (participants names, contact details, socio-demographic data), and the list linking them with the participant ID numbers, were stored within password-protected folders on a secure drive on NHS Trust computers, accessible only to the research team. Data will be retained and archived in accordance with the Research Governance Framework, East London NHS Foundation Trust Record Management, and Information Management and Technology (IM&T) security policies.

## Results

In total 29 service users and 16 clinicians used the DIALOG+ intervention over a 3-month period and all of them were invited to take part in a semi-structured interview. Of these, 19 service users and 15 clinicians were actually interviewed about their experiences. One interview was terminated early and the resulting transcript removed from the analysis due to insufficient data. Eighteen transcripts in total were included in the analysis. The recruitment flow is outlined in Table2.

**Table 2:** Recruitment flow

Participants	Number of participants approached	Number of participants interviewed	Interview Location	Mean average duration of the interviews (range)
**Service Users**	28 service users who received DIALOG+ intervention for 3months	**19interviewed ** 6 declined 2 not contactable 1 unwell	Service users homes, community services or premises of research departments	25min (1250.5)
**Mental Health Professionals**	16 mental health professionals who delivered DIALOG+ intervention for 3months	**15 interviewed** 1 left service	Mental health professionals work premises	31min (1458)

The socio-demographic and clinical characteristics of the participants are presented in Tables3 and 4. The frequency of delivering DIALOG+ sessions during the 3-month intervention period varied from once per month to once per week depending on the clinicians job role and the needs of individual service users. The clinicians reported that some of the service users they worked with received less than the suggested minimum number of sessions due to them cancelling or not attending appointments. Out of 19 service users who agreed to be interviewed, ten received the DIALOG+ sessions monthly, four fortnightly, and four on a weekly basis.

**Table 3:** Socio-demographic characteristics of service users

Characteristics of participants	***N***=19
**Sex**	
Female	13
Male	6
**Age** ***(mean; range)***	45(2568)
**Marital Status**	
Single/unmarried	10
Married / Co-habiting	6
Separated/Divorced	3
**Education**	
Primary education or less	1
Secondary education	6
Tertiary/further education	3
Higher education	9
**Employment status**	
Employed full/part time	5
Voluntary employment	3
Unemployed	7
Retired	4
**Living situation**^a^	
Living alone	8
Living with partner/family	9
Living in shared accommodation	1
**Migration status**^b^	
Born in UK	16
Born outside UK	2
**Ethnicity**	
White British & Irish & Other	14
Black British African & Caribbean & Other	4
Asian British Indian & Bangladeshi & Pakistani & Other	1
**Length of treatment for depression** ***(mean; range)***	6.5 (037)
**Type of clinicians currently seen for treatment of depression**	
GP	9
Primary care nurse	3
Care-coordinator	10
Psychiatric Nurse	8
Psychiatrist	15
Psychologist /Psychotherapist	5

^a,b^One value missing

**Table 4:** Socio-demographic characteristics of mental health professionals

Characteristics of participants	***N***=15
**Sex**	
Female	10
Male	5
**Age range**^**a**^	
<35	3
3549	5
5065	6
**Ethnicity**^**b**^	
White British & Irish & Other	12
Black British African & Caribbean & Other	1
Other (Latin American)	1
**Length of experience of working in mental health**^**c**^ *(mean; range)*	17.4 (4.536)
**Professional background**^**d**^	
Community psychiatric nurse	9
Psychiatrist	1
Senior Support worker	1
Occupational therapist	1
Social worker	1
Psychologist	1
**Current professional roles**	
Care-coordinator	3
Clinical psychologist	1
Consultant psychiatrist	1
Primary care mental health nurse	1
Primary care liaison mental health practitioner	1
Community psychiatric nurse & recovery worker	6
Senior mental health triage nurse	1
Senior support worker	1

^a,b,c,d^One value missing

### Themes

The analysis resulted in five overarching, and interrelated, themes about the experience of using the DIALOG+ intervention: DIALOG+ Structure; Therapeutic Communication; Reflecting and Monitoring; Empowerment and Powerlessness; and The Impact of Technology. Each of the five themes reflects significant aspects of the participants experience of using the intervention, and illustrates how these aspects could be seen as both positive and negative, sometimes by the same participants. The presented themes are rich descriptive overviews of a variety of their views and experiences. A summary of the overarching themes and subthemes is provided in Table5. The additional service users and mental health professionals quotes are included in Table6.

**Table 5:** Summary of themes

**DIALOG+ structure**	
**Clarity & focus**	
**Getting a whole picture**	
**Constraints of the format**	
**Therapeutic communication**	
**Topics Reminder**	
**Opening new conversations**	
**Disrupting the flow**	
**Losing personal touch**	
**Reflecting and monitoring**	
**Reflecting on strengths & difficulties**	
**Mapping change**	
**Quantifying feelings**	
**Empowerment and powerlessness**	
**Taking ownership of recovery**	
**Setting & achieving goals**	
**Becoming discouraged**	
**Feeling scrutinised**	
**The impact of technology**	
**Transparency & accessibility**	
**Therapeutic aid & hindrance**	
**Technical competence & issues**

**Table 6:** Service users and mental health professionals quotes

**DIALOG+ structure**	
**Clarity & focus**	
**T1P08_service user** our sessions werent really aimed at anything, it was just me talking about how my last weeks gone and thats it, whereas this one was more targeted at what problems I have and what issues I have and how we combat those issues and what well do to move forward from those issues. So I think that was quite good	**T1C07_clinician** And it helps them to keep focused on the one topic in hand as well. Sometimes if youre just having a conversation with a person they can tend to drift to another topic. At least with this it kept them focused on, OK, my physical health, lets just talk about that. So it was good on that score.
**Getting a whole picture**	
**T1P15_service users** its better really because then your therapist can get a whole picture of your life as opposed to only certain aspects of it, which can all contribute on your mental health wellbeing, because they might be causing a certain amount of the issues	**T3C01_clinician** And that would be helpful to cover, to look at somebodys life in a holistic way like that I think is great and it kind of reminds you that actually theres other domains to this persons life that if there were an improvement actually it would have a knock on effect on their mental health.
**Constraints of the format**	
**T3P01_service user** with (*care co-ordinator*) we only have half an hour session, so it can be a little bit, rather than spending time, I felt a little bit rushed to answer some of these. Rather than when you sit down, and you physically go through them and care co-ordinator will say hows this, hows that.	**T3C02_clinician** But because I have had an established relationship and we already had set ways of working it kind of got in the way of things that wed already found to work ... So some of the questions werent relevant or they were too broad for us
**Therapeutic communication**	
**Topics Reminder**	
**T2P10_service user** once or twice I suppose when we were going down, I thought, oh actually we havent talked about that, and it sort of jogged memory a few times. So thats again where I think the headings are valuable because they do, they are quite a useful jab in the ribs almost	**T1C12_clinician** I think the app is brilliant, its very good, it helps clinicians because sometimes we get complacent on what sort of questions we ask patients and so it helps to explore more on issues .
**Opening new conversations**	
**T1P08_service user** I think it wouldnt have come up because I wouldnt have thought about it. But having these specific questions make you think more so then youve got more to add to the conversation	**T2C05_clinician** This particular patient seemed to see its logic straightaway and just go with it and find it pretty helpful and it did lead us into quite useful discussions I hadnt had with him before so that was all good
**Disrupting the flow**	
**T3P03_service user** I think it didnt really cover the way that I feel and my problems really I just felt like it was ticking the boxes really rather than an in depth conversation.	**T3C02_clinician** It disrupts the flow of a conversation because youre having to stick to topics one at a time whereas actually quite often they all mush into one. Medication affects motivation and sleep affects everything else. So its just kind of, its, yeah, it doesnt really take into account that which I found frustrating.
**Losing personal touch**	
**T3P01_service user** I think it is a bit impersonal, a little bit. Because sometimes when youre in a mental health meeting, you go in and speak to your mental health worker, it can make it a little bit, well youve got to stick to this.	**T2C03_clinician** a lot of work that we do is personable and takes a lot of just general chatting whereas this is like . some patients need the focus and other dont need that focus, you need to have more of a personal edge to things. its less personal and I think people with depression () they want to feel that youre listening to them as opposed to collecting scores.
**Reflecting and monitoring**	
**Reflecting on strengths & difficulties**	
**T1P15_service user** it makes a refreshing change to talk about something thats good as opposed to everything doom and gloom and no hope and light at the end of the tunnel. So its nice to talk about the good things as well as the bad things.	**T2C01_clinician** following the four step thing actually made the patients think about what they had scored a little more carefully than they might otherwise have done necessarily. Just that little extra . helped them to think a little bit more about this even if didnt necessarily achieve what they said they did it stimulated them to think about what could be going on here, what could be doing to try and make a difference even if I cant do it.
**Mapping change**	
**T3P02_service user** It feels often quite a bit of backsliding, but you looked and thought, oh, actually, that wasnt as bad as I remembered it to be, or oh actually, that was quite a good week. And, oh damn, this week is not very good, but last week was fine () I think it would be great for mapping progress.	**T3C01_clinician** you can literally show a patient, look how things have changed for you. Or even if things havent changed so much, it might be that they had a, maybe a good week or a good month, whatever it might be where you can talk about, well, look how well you did here. I know it feels like everythings bad now but you had that really good time there and what were the things that were contributing to that? So again I think that could be really helpful.
**Quantifying feelings**	
**T3P04_service user** Sometimes its hard because if youre trying to think over the week, obviously, some days are different to others so it can be quite hard to actually pick a number of what it is overall.	**T2C02_clinician** for them it was hard to sometimes say, well, Ill give it a four then rather than a three, without really thinking through, well, what does a four mean compared to a three sort of thing. So I think they were just, it was just throw numbers at me a little bit really. So I dont think the rating thing was marvellously helpful for the patient or myself.
**Empowerment and powerlessness**	
**Taking ownership of recovery**	
**T1P07_service user** previously, as I said, I would just be sitting down and waiting for him to bring the idea and so that I will bring conversation. But with this it helps to remind me and also help me to speak my mind on what I want to tell him	**T1C09_clinician** And also they get more involved I think they have to think about how, what they can do to make an improvement, whereas otherwise . a lot of people tend to put it on the professional, they just feel that theres nothing they can do and here its very much focusing on, theyre taking a bit more responsibility as well, and expectation so they can do something or the family.
**Setting & achieving goals**	
**T2P02_service user** I think the helpful element was again to go back to my experience, was the goals it gave me on physical health which built me, started walking, were very helpful in improving my health and fighting depression. I felt quite combative and as though I was fighting the depression by virtue of the fact that I was walking.	**T3C03_clinician** I think again its about specific goal setting, and I think a lot of the patients with depression struggle with functionality, and I think it does break down the different areas in their life, and I think that foundation is really good around recovery.
**Becoming discouraged**	
**T1P08_service user** So like I picked two from the topics for 1 week, and the next week, because those two hadnt been resolved, Im still on those two and so its kind of like a vicious circle and not being able to do the other lot.	**T3C01_clinician** job situation for some people that might be a bit of, something thats contributing to their depression That might be something thats quite a trigger thing for them to say, well, no, still no job. Come back to that week by week. Its like, oh gosh, hows that going to feel then just revisiting that.
**Feeling scrutinised**	
**T2P03_service user** I found that quite hard to judge the level of where Im on those topics. After the first session and we come back to the second session, I felt the need to rate it higher. Just so I could see improvement even though I didnt feel like that.	**T3C05_clinician** Then we introduced the DIALOG system and he dropped out... maybe the expectation of in a conversation lets set some goals, and the app was like quantifying how much theyd improved. So if they felt a little bit guilty, like, oh, Ive got to go and see him now and I havent really improved my relationships and things, and he just thought, oh, Ive got bigger things, whatever
**The impact of technology**	
**Transparency & accessibility**	
**T1P10_service user** Well, before hed ask me all these issues but there was no way that he could make a comparison hed have to spend a lot of time to analyse what notes have been written last time and then hed have to read them when I came the next time to make the comparison that have I moved forward or not. But with this it was instant.	**T1C09_clinician** I think also with noting it down on the tablet it makes it easier to remember the actions you could just go back and you remind yourself what you put down as actions. So thats good.**
**Therapeutic aid & hindrance**	
**T2P09_service user** I think again, yeah, just having a visual representation of how certain things feel at the time, kind of allowed me to explain it a bit more.	**T2C03_clinician** ..,youre trying to establish a relationship with someone to help them get better and thats normally focused on talking and showing empathy and understanding, its quite hard to do that when youre typing in or when youre pressing numbers.
**Technical competence & issues**	
**T3P04_service user** I mean it was quite frustrating because it was quite slow and unresponsive, if I wasnt necessarily having *a* good day I didnt really have the patience to be working with it."	**T2C02_clinician** certainly the first patient was pressing the buttons for me because I got confused and so he seemed to know a bit more about what he was talking, so I relied on him which I suppose is good and bad really. I suppose he owns the session by doing that

### DIALOG+ structure

Using DIALOG+ brought more structure into regular clinical meetings for both service users and mental health professionals, introducing a clear process to follow. Rating and reviewing 11 areas of the DIALOG scale helped participants to identify the most pressing issues to focus on, while the 4-step approach facilitated exploring solutions for the selected areas. In comparison, past sessions often lacked specific purpose, and could be overwhelming when multiple issues were presented.

* sometimes the sessions can run on and on and on and you end up getting nowhere. So I thought having a device with an app that gives specific areas that you want to talk about, giving the person the choice of what the questions are, like what they wanted to talk about, I thought that was really good and could hone in and keep the session more specific and help us to work towards goals.*

**(*ID: T2C03_clinician*)**

Considering different areas of life, rather than focusing on symptoms only, helped service users and clinicians to work in a more holistic manner to better understand how mental health interacts with and impacts on other areas of life. This broader approach enabled service users to identify life areas they were more satisfied with and could therefore draw strategies from.

*I think also its good because obviously mental health does affect a big range of things. And I think its a good way of looking at all the different things rather than just discussing mental health as one thing if that makes sense ( ) because I didnt realise how some of these different things are affected by my actual mental health. So, for me it was a bit of an eye opener.*

***(ID: T3P01_service user*)**

Although most service users and clinicians acknowledged the benefits of the structure afforded by DIALOG+, some found the format too rigid and prescriptive, preventing them from talking about all the topics they wanted to address, or talking about them in sufficient detail. Others felt that the app was imposing unnecessary or irrelevant topics to be discussed, with some suggesting that a more personalised approach may be required to stop vulnerable service users becoming overwhelmed. A few clinicians struggled to complete the intervention within the allocated time, sometimes significantly prolonging the sessions.

*I think it was quite good but it didn't always leave time for me to say what I was struggling with and what I needed help with.*

***(ID: T2P10_service user)***

Service users and clinicians who had a pre-established therapeutic relationship often found sessions somewhat repetitive as they had to revisit previously discussed issues. However, they did acknowledge that the intervention would be a helpful tool to build the therapeutic relationship with those newly referred.

### Therapeutic communication

Some service users and clinicians found that using DIALOG+ improved the way they communicated in their meetings. The app prompted them to cover a range of domains and helped them remember which issues to address. It also reminded them what was agreed in previous meetings, and this was seen as particularly useful for those service users who struggle with memory and/or have less frequent sessions.

*And I think it would help a patient, especially patients who are not very confident or cant remember what issues there, so this sort of triggers them, oh, my medication isnt right or something. So when you read something it sort of comes back to you, and sometimes, you go shopping and you dont write things down, you buy everything else but you forget what you went out for ( ) it works the same way, it just prompts you *

***(ID: T1P10_service user)***

Both the DIALOG scale and the 4-step approach enabled the participants to expand the scope of their conversations by opening up new topics or exploring issues in more detail or depth. Rating the life areas with the DIALOG scale was also seen as a less intrusive way to initiate conversations on sensitive topics or with service users who were not confident enough to directly express their thoughts and feelings.

*I found the four stepped approach, when you break it down, I found that really helpful because I think some of the questions that, normally, I might not have asked of a client like what can you do, what can I do, what could others do (...) That helped me to have some conversations with patients Ive actually known quite well for quite a long time that we havent explored before.*

***(ID: T3C03_clinician)***

Conversely, others felt that using the app impaired the natural flow of communication, making it more confined to the specific areas included on the scale and less likely to spontaneously move in other directions. It was argued that free-flowing conversations can be more effective particularly with service users who struggle to keep focus.

*I didnt feel I was having a real conversation with her. I felt I was just going through ticking the boxes type of thing.*

*(****ID: T3P03_service user****)*

Introducing DIALOG+ into the pre-established therapeutic alliance sometimes made the conversations feel artificial, however, some clinicians also reported being able to adapt the intervention to their personal style.

### Reflecting and monitoring

Using the DIALOG+ app, in particular rating satisfaction with the life domains, encouraged service users to reflect more on both their strengths and difficulties, the impact they have on their life, and how to make changes. This process of self-reflection sometimes continued between the sessions. The satisfaction ratings also provided quantifiable information which contributed to clinicians insight, enabling them to explore discrepancies with service users verbal explanations.

*Well, it certainly gave me time to think about those areas where you dont think about when you have to answer that, what is my family life from one to ten, and then you sort of think about it, is it really that bad or is it really that great? And so I think it gives you a tool to analyse yourself before you answer.*

**(*ID: T1P10_service user*)**

The ratings on the scale were visually displayed on the tablet and could be compared across sessions. This feature was particularly useful for therapeutic reflection, enabling service users and clinicians to monitor changes over time and identify areas of improvement or deterioration. Improving scores provided encouragement to service users, while worsening scores prompted discussions about the possible causes of deterioration and how to avoid them in future. Seeing improvement sometimes enabled service users to realise that they were doing better than they thought.

*The clients themselves could have a look and think, yeah, last month I was having this issue but this month Ive been able to resolve that. They can feel some sense of achievement with themselves, being able to look at the information for themselves and I think its very useful like that.*

***(ID: T1C07_clinician)***

The visual representation of the data could also be a reminder about the lack of improvement. Seeing low satisfaction values was an uncomfortable experience for one service user, while another saw it as potentially upsetting.

*OK, so Im a bit split on this. I think its, like I said, its a really good thing because you can see how youre improving or youre not improving and then how you need to focus on these different things more than others that are listed. Whereas also ( ) actually physically seeing it measured, for me was a little bit... I dont know, it brings home a little bit how bad some of these actually are, and how bad at times my actual mental health is, and how it affects me.* ***(ID: T3P01_service user)***

Some clinicians observed that the service users they worked with had difficulties in recognising improvements even when they reported higher satisfaction scores. They emphasised that rapid progress is unlikely to happen in service users who have long-term conditions, and that difficulties in acknowledging positive change may be a feature of chronic depression.

* there wasnt ever much change and with the one where there was a change she couldnt really accept that there was that change then. It was more oh, thered been a mistake or I don't know why its higher or lower this week.*

***(ID: T3CO4_clinician)***

Several service users and clinicians did not find the DIALOG scale easy to use. Some found quantifying feelings difficult whilst others argued that the scale was not sensitive enough to capture small changes occurring between frequent appointments.

### Empowerment and powerlessness

DIALOG+ increased the service users confidence to engage in decision making about their care and helped them regain the sense of ownership of their recovery. Some felt that they were more listened to and their views acknowledged by clinicians. Focusing on just a few specific areas and setting actions to complete before the next session made progress achievable and measurable and, in turn, improved motivation.

*So DIALOG+ was like involving me to let me speak up and look me in my face to say that theyre interested in what Im saying ( ) it helped me to have a voice and to be heard *

**(*ID: T1P09_service user*)**

Some clinicians felt that DIALOG+ made the treatment decision making process transparent, demonstrating to service users how to co-design their own care plan. The physical handing over of the tablet to the service users was symbolic of clinicians trust in them and their sharing of responsibility.

*It gave focus. It gave ownership to the client because the client would be out there seeing where we are going, where we are coming from. Especially after the third session the client understands the framework ( ) as a practitioner this gives me even more ammunition actually to work with a client, to show and help the client to see that he can be the owner of his co-ordination of care and the tablet does contribute to that.*

**(*ID: T1C08_clinician*)**

In contrast, some service users struggled to come up with actions that could bring about change or were worried about how little they could actually do. They felt scrutinised about making progress in life domains and completing agreed actions they did not feel able or willing to accomplish. These observations were mainly reported by the clinicians with some of them emphasising that this pressure could potentially lead to disengagement. Focusing on too much change too soon may feel threatening to service users with chronic depression as it invalidates withdrawal behaviours they find safety in.

Service users were sometimes discouraged by seeing a lack of improvement over time in specific areas, especially if they felt that moving forward was contingent on decisions made by professionals or services. Revisiting these topics from session to session without progress or resolution made the meetings repetitive and disheartening for both parties. Clinicians were occasionally limited in what help they could provide which was disappointing for some service users.

*Sometimes I felt it could be pointless because there wasn't any obvious answers as what could be done to make it better and it was repetitive from one session to the next, like, I'm still feeling rubbish at my mental health and all that could be added for an action was to keep engaging with support and I don't know how useful that was. But on other topics, running through the steps I think were good.*

***(ID: T2PO5_service user)***

### The impact of technology

The participants highlighted technology-related benefits including easily accessible information, the visual display of routinely collected outcome data, and the paper-free nature of the intervention. Recording satisfaction ratings and agreed actions on the tablet contributed to service users perception of greater transparency, accountability and even safety of personal data. Some suggested that the app should be more interactive to allow service users to electronically update clinicians on their achievements or any changes to the planned actions.

Visually displaying the satisfaction ratings on the tablet screen and being able to instantly compare these ratings across sessions was seen as more effective than verbal explanations.

* sometimes just in conversation you dont necessarily really realise how you feel or how serious or how bad a certain thing can be, and then if you can ( ) see it in front of you, and actually rate it on a scale, you can get a better idea of how it actually is.*

***(ID: T2P09_service user)***

However, some clinicians reported that the focus on navigating the app, via a tablet, reduced faceto-face contact and opportunities to observe service users or show empathy. It was felt that this process took away some spontaneity and rapport they had previously, with some explicitly stating their dislike of using technology in a therapeutic context.

*I want to say positive things but it made it harder because youre both staring at something together and youre kind of sat alongside ( ) its harder to pick up on facial cues or things that are going on. So theyre saying one thing but actually theyre showing something else. It kind of takes away that element of it.*

***(ID: T3C02_clinician)***

The tablet sharing aspect of DIALOG+ meant that the clinicians had to sit close to service users. Two clinicians were uncomfortable because of reduced personal space or safety concerns, while a third felt that this proximity made their client anxious.

Technical difficulties such as slow-loading software and the need to repeatedly re-enter passwords after screen timeout were frequently reported by the clinicians and service users, sometimes interrupting the flow or prolonging the sessions. The received training was positively appraised, however the time gap between being trained and actually using the app in practice sometimes contributed to insecurities with the procedures.

* so this is during a session with somebody to say, oh damn, its timed us out ( ) so it wasnt a flowing discussion because I had to keep going, oh, hang on, Ive got to find the password to get us back. So I suppose it didnt help the therapeutic milieu if you like.*

***(ID: T2C02_clinician)***

In addition to experiencing technical issues, almost half of the clinicians had some doubts about their competency in navigating the app. They worried about service users noticing their insecurities, and the potential negative impact this could have on the interaction, particularly with those who were highly distressed.

*Yeah, and I wanted it to work but I was a bit worried about making myself look a bit of an idiot by not knowing how it was working which is why I tried to go through it quickly before I went to see her so I could at least sound like I knew what I was talking about. But when youre in a situation with someone crying and youre trying to work your way through this app its quite [difficult]* ***(ID: T2C03_clinician)***

### The preferences for future use of DIALOG+

Out of 18 service users, 15 expressed interest in continued use of the intervention, 2 declined and 1 was neutral. Of those who declined, one felt that the intervention was needlessly prolonging sessions, whilst the other was frustrated by the lack of change over time in certain domains.

Out of 15 clinicians, 13 were interested in its future use. One who declined found the app-based intervention too restrictive but was still interested in using a paper-based version. The other clinician felt that the DIALOG+ intervention would be unnecessary as his practice already incorporated the same principles.

## Discussion

The acceptability and relevance of the DIALOG+ intervention for people with chronic depression was investigated by eliciting the views of service users and mental health professionals who tested it for 3months in community-based settings. Thematic analysis identified five overarching themes which highlighted both positive and negative aspects of using the DIALOG+ intervention within the same themes: DIALOG+ Structure; Therapeutic Communication; Reflecting and Monitoring; Empowerment and Powerlessness; and The Impact of Technology. Most participants expressed interest in continuing to use the intervention, highlighting overall acceptability. The themes in the present study were consistent with those identified by Omer et al. [20] who investigated the mechanisms of action for the DIALOG+ intervention in the community treatment of service users with psychosis. The themes presented as part of that work (Comprehensive structure; Self-reflection; Therapeutic self-expression; and Empowerment) map onto the themes presented here. This shows that broadly, the experience of using DIALOG+ is similar across diagnoses.

DIALOG+ improved the therapeutic communication for some service users and clinicians in our study, as it prompted them with topics that may not have been ordinarily addressed in the past. These prompts broadened conversations or opened up new ones, sometimes making it easier to talk about sensitive issues. In addition to similar findings in Omer et al. study [20], this is consistent with past research showing that completing a simple communication checklist before appointments led to improved quality of clinical communication and changes in treatment [21].

Some service users actually reported that using DIALOG+ enabled them to be more vocal about their needs. The clinicians felt that the intervention made the process of designing care plans more transparent and facilitated co-production of the treatment plans. Similar observations were reported by the clinicians involved in the study of the implementation of a mobile digital care pathway tool (CPT) [22]. Clinical orientation towards increasing involvement of service users in decision making has been shown to improve satisfaction with the care decisions [23] and reduce decisional conflict [24].

Crucially, the importance of service user empowerment in all areas of life, and not just mental health, has been widely recognised [25]. The comprehensive nature of DIALOG+ may facilitate an improving sense of competence across different life domains [20]. Monitoring satisfaction ratings on the DIALOG scale by comparing them across sessions was a popular feature as it allowed individuals to identify and reflect on causes of improvement and/or deterioration. Previous studies have established that evaluating treatment progress using standardised assessments and providing feedback to therapists and/or service users has the potential to improve outcomes [26], including reducing depressive symptoms [27]. On the other hand, chronic depression may lead to a deeply entrenched sense of hopelessness and fear of change in sufferers [28] which could potentially make them reluctant to perceive or acknowledge progress. Consistent with this, some clinicians described how service users sometimes reported increased satisfaction ratings but without any subjective feeling of improvement, leading to a difference in perspectives. Seeing deteriorating satisfaction ratings or not being able to complete the agreed actions could potentially reinforce negative feedback that depressed individuals may be particularly sensitive to [29]. Caution should therefore be exercised not to pressure service users with chronic depression into making changes too fast, or setting behavioural goals that are too large, as this may feel invalidating to them [28].

Service users perception of their needs may differ significantly from the views of clinicians who treat them, with the severity of depression having been shown to predict lower agreement [30]. Most service users and clinicians in our study reported that the structure of DIALOG+ facilitated easier identification of the most important issues to work on and subsequently helped to set relevant actions.

DIALOG+ made the assessment and comparison of satisfaction with life domains instantly available and easily accessible through the use of an app. A number of participants highlighted the positive impact of the visual display of satisfaction ratings on service users insight, which was sometimes more effective than verbal communication. The use of technology, although not the main feature of the intervention, may enhance the therapeutic effects [20, 31, 32]. On the other hand, some clinicians expressed concerns about the impact of technology on therapeutic alliance and interpersonal dynamics. Similar worries were previously reported by the clinicians testing DIALOG+ with service users with psychosis [15] and those implementing a digital mobile CPT tool [22]. Such views amongst clinicians are well known as barriers to the adoption of technology-enhanced services [33, 34]. More comprehensive training resources and offering ongoing support for clinicians in their use of DIALOG+ could potentially address many of the negative aspects reported by service users and clinicians. For example, enhanced training could improve clinicians confidence with using technology or ensure that they are not too prescriptive in the delivery of the intervention, thus avoiding a tick box exercise effect and using the intervention as a framework to work from rather than a strict procedure to stick to.

### Strengths and limitations

A main strength of the study is that we elicited the views of both service users and mental health professionals of varied professional backgrounds in community care settings, including those based in primary care services, across urban, semi-urban and rural areas in England. An advisory panel of service users and carers were actively involved in designing the study, developing the topic guides, and discussing the evolving themes. One service user researcher was involved in analysing the interview data and naming the themes.

The study also has some limitations. We did not specifically ask about the participants confidence with technology or level of computer affinity, which can be a significant determinant in engagement and satisfaction with technology-supported interventions. The sample may have been biased towards those interested in technology, with the clinicians potentially choosing service users from their caseloads who they thought were good for or interested in the intervention. Despite the absence of a specific question about confidence with technology, almost half of the clinicians expressed either doubts about their IT competency or reservations about technology-supported treatments. However, most of them were interested in continuing to use the DIALOG+ intervention, with only one clinician specifying his dislike of technology as a reason for not being interested in future use. Taking into account the wide age range of the participants and a variety of views and experiences they reported, we believe that our sample encompassed clinicians and service users with a range of technological abilities and preferences.

## Conclusions

The findings indicate that DIALOG+ was broadly acceptable for service users with chronic depression and clinicians from different clinical settings, albeit with some caveats. The concerns raised by both clinicians and service users, such as the overly prescriptive structure and worries about technological competency, could be partially addressed with improved training, ongoing supervision and increased familiarisation of the DIALOG+ app. The clinicians worried about the impact their insecurities about using technology may have had on service users perceptions of the intervention and their own competence. This finding highlighted the need for top up training sessions for clinicians and for continuing technological support. Similar issues of clinicians confidence and perceived ability influencing implementation of the digital CPT tool, as well as the need for ongoing support were reported by Pithara et al. [22].

## Supplementary Information

**Additional file 1.** DIALOG+ app_DIALOG scale.

**Additional file 2.** DIALOG+ app_Comparison feature.

**Additional file 3.** DIALOG+ app_4 step approach.

**Additional file 4.** Service users quotes: Quotes from the interviews with service users.

**Additional file 5.** Mental health professionals quotes: Quotes from the interviews with mental health professionals.

## Data Availability

The datasets used and/or analysed during the current study are available from the corresponding author on reasonable request.

## Abbreviations

NHS: National Health Service
TACK Programme Grant: Tackling chronic depression Programme Grant
NICE guidelines: National Institute for Health and Care Excellence guidelines
IM&T: Information Management and Technology
CPT: Care pathway tool
