# Supplementary figures and images for: Acceptability of a technology-supported and solution-focused intervention (DIALOG+) for chronic depression: views of service users and clinicians
Source: BMC Psychiatry. 2021 May 20;21:263. doi: 10.1186/s12888-021-03256-5 (PMC8139109; doi:10.1186/s12888-021-03256-5)

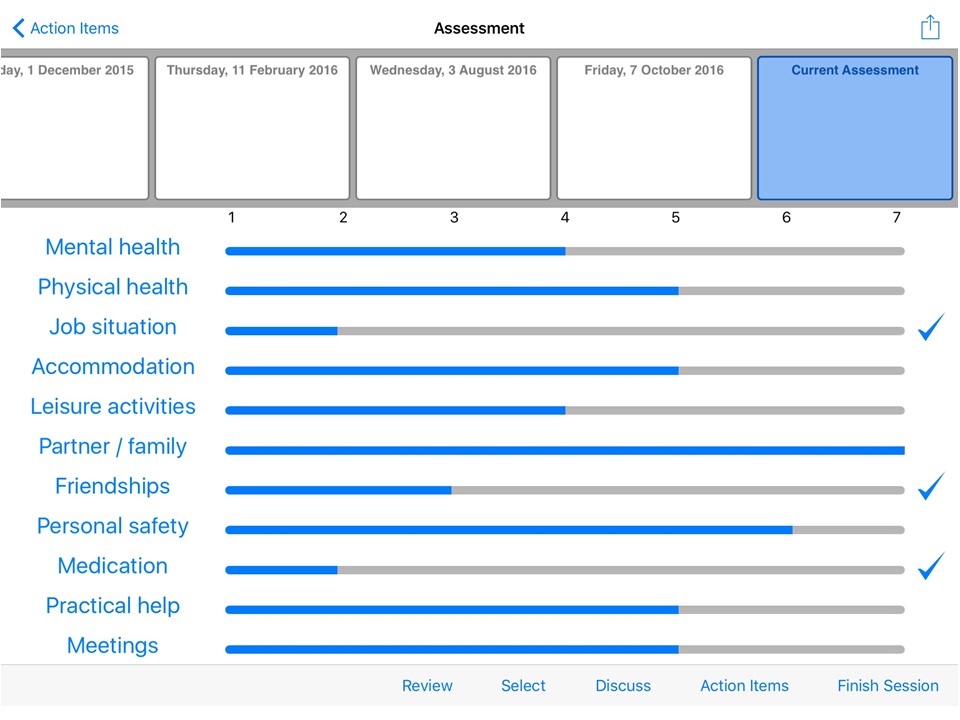

Supplement: Supplementary file 1 — Additional file 1. DIALOG+ app_DIALOG scale. [file 12888_2021_3256_MOESM1_ESM.jpg]

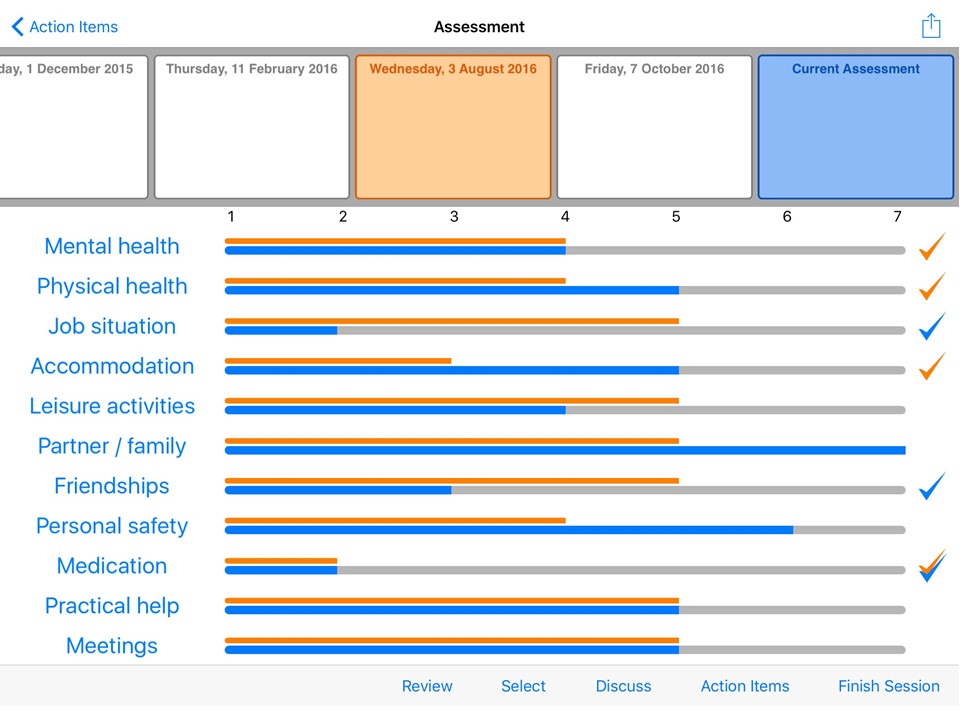

Supplement: Supplementary file 2 — Additional file 2. DIALOG+ app_Comparison feature. [file 12888_2021_3256_MOESM2_ESM.jpg]

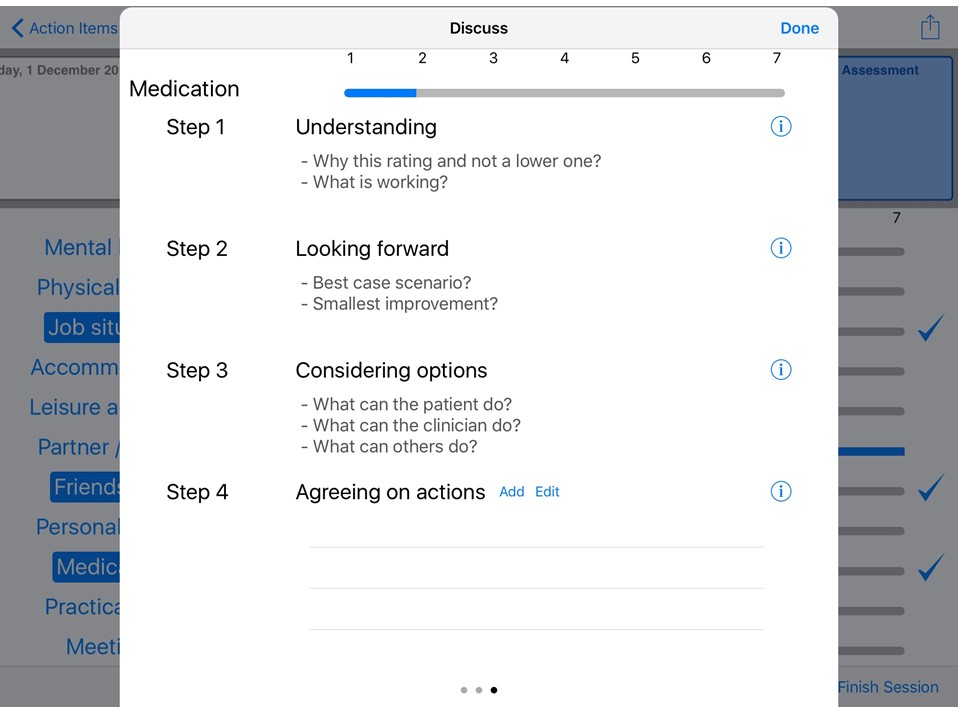

Supplement: Supplementary file 3 — Additional file 3. DIALOG+ app_4 step approach. [file 12888_2021_3256_MOESM3_ESM.jpg]
