# Supplementary material for: Acceptability of a technology-supported and solution-focused intervention (DIALOG+) for chronic depression: views of service users and clinicians
Source: BMC Psychiatry. 2021 May 20;21:263. doi: 10.1186/s12888-021-03256-5 (PMC8139109; doi:10.1186/s12888-021-03256-5)
Supplement: Supplementary file 4 — Additional file 4. Service users quotes: Quotes from the interviews with service users. [file 12888_2021_3256_MOESM4_ESM.pdf]

SUPPLEMENTARY MATERIAL: ADDITIONAL FILE 4

SERVICE USERS' QUOTES

THEME 1: DIALOG+ STRUCTURE

T1P07_Service User

Quote1

"…with this it's like there is a point being set down and a process which needs to be followed …and easy to discuss.….. I want to talk about this, I want to talk about this. … it's like give me guidance on what to do and what to talk about … and this helped because previously I will just be looking at him."

Quote2

"…I find it difficult to cope but when there is a structure and plan it helps me to know… Actually sometimes my coping strategies is going out, doing things myself and, those, I don't like it, so it's like when this is in place it helps me… I know that this is what I'm to do … and it's done so I use it."

T1P08_Service User

Quote1

"…it's made … the appointment more structured and we've been able to discuss certain areas that I've chosen. Obviously I would have liked more time to look at different areas but I think it's good that we could structure it more…"

T1P10_Service User

Quote1

" And you can focus on what is stopping you from moving forward and to get your confidence back I suppose. So you can work out the things that aren't working and that's what (*name of care-coordinator*) did …So it's helpful to the care co-ordinator as well because then they know …. which area to help with otherwise they're lost as well."

Quote2

"…sometimes just giving, taking medicine doesn't give you the option to think about things and look at yourself at different, like my work … my health, my family and it sort of brings it in front of you that, whereas before it was just the whole thing. You just think that it's the end of the world but it gives you a chance to recognise different aspects of your life …"

T2P02_Service User

"I think the good thing about is that it gives a structure and it's a simple structure it gives objectivity and measurement and it also gives, makes it very, gives clear goals to what's going to be achieved very simple, clear goals. "

T2P05_Service User

"So, previously there wouldn't always be a topic or a direction to the meeting, I'd come to my meetings and it would just be, how are you? What's been happening, generalised, you know and if there was a problem that would be explored further… but using this, what do you call it? DIALOG… The meetings felt more structured and everything would be covered, really."

T2P09_Service User

"…I felt like it made my sessions a bit more structured and it allowed us to identify where I was specifically feeling low kind of thing, in what areas and how I could improve them."

T2P10_Service User

" I think that it's a good way forward, with all the kinks sorted out , because it gives, it does give focus to the session that you have and it gives you the space to explore things that you didn't know might be affecting you. "

T3P01 Service User

Quote1

"… we focused on maybe one or two points depending on what were the worst in relation to my scores. And I think, like I said before, we did focus on those that are affecting me the most, so when I actually came out the session, rather than being completely overloaded, I could cope with the small, little bite-sized different things."

Quote2

"… we discussed the bad things but then we then looked at options to make those better, and obviously by agreeing on actions at the end, you go away with goals to focus on. So for me I think, rather than going off on a tangent and not getting as much out of the session as I could, by sticking to some sort of framework, I think was better."

T3P09 Service User

"I've been seeing her for quite a long time now so I suppose she probably listens to me fairly well … But if I was a new patient to her then, yeah, I would expect this would cut the amount of time it would take to understand me and what it was like going forward."

THEME 2: THERAPEUTIC COMMUNICATION

T1P07_Service User

Quote1

"…sometimes I want to talk but it's like in my head...I just keep quiet. Does he really know what I want to say? I'm waiting for him to ask me. Are you OK? Yes, I am. But when he brings that *(DIALOG+)* that helps me to know what we need to do and what we need to talk about."

Quote2

"But when this came in, it's like they know what to do and what to put in place and it also helped me to know what I want to discuss as well with them. …So I feel this should be, and this should stay in."

T1P10_Service User

Quote1

"So sometimes I'd go in and I'd forgotten what was the issues that I had last time but (*name of clinician*) would bring them up, that you were having trouble overcoming these aspects and then it sort of clicked my mind then… And so it definitely helped both of us…"

Quote2

"It was highlighting the areas and then talking about them and then you'd say, oh yeah, I'm really not happy with my kitchen or the heating, and that's just examples, but it sort of triggered those where I wouldn't talk about …. with *(name of clinician)."*

T1P15_Service User

"… if you did feel suicidal or something and you don't want to voice it, maybe it's easier to put it on a bit of paper than actually voice it to somebody…Not everyone's the same but I think I would find it easier to put it on paper than vocalize it myself, yeah…"

T2P10_Service User

"And it was interesting to talk about different subjects that I hadn't really been thinking about or if I had they were at the back of my mind"

T3P01_Service User

Quote1

"… sometimes you need to sit down and, like I said, you can't always, as you're talking about some things, it does tend to lead onto other things. Whereas we had to stick and then we obviously went through and measured how these different things affect, but we didn't go into detail as in things like what medication

I'm on, how it affects me on a day to day basis, or my job situation or accommodation. It was a bit tick boxy for me…

Quote2

"… sometimes it's difficult to stick to specific points and I think sometimes these can maybe not necessarily go in that order, because you might go through something and think oh actually, I need to go back. But like I said I do think this is a good framework for getting the most out of your session."

T3P02_Service User

Quote1

"I think sometimes when you're seeing somebody fortnightly or monthly, you slightly lose … you don`t always get, have a very good overview on where discussions have been. So I think if you were able to look at that and think, oh yeah, actually that was quite, you know, quite constructive…"

THEME 3: REFLECTING AND MONITORING

T1P08_Service User

"I think it has made a difference to our sessions. It's made us more aware of what we both think and what we're both doing during the weeks that … we haven't seen each other."

T1P10_Service User

Quote1

"Before it wasn't like that. Before it was like just you have a chat and he writes down what I feel, what I'm feeling and that was it. So there wasn't sort of a picture to see that how I was last month and how I am this month…."

Quote2

"… I could monitor what my mood swings were the previous time I met him and try to pinpoint why they were in that four week timeframe and try to work out with (*name of clinician*) … how to avoid getting into those areas."

T1P15_Service User

Quote1

"… I did definitely find it useful because you could look back and see if there's any progress or worsening of how you feel and how helpful it had been."

Quote2

”I don’t think I would have been discouraged. I think I would have been quite disappointed in myself and probably quite alarmed at it, especially the mental health one and the personal safety one. I think they would be the two main ones that would alarm me the most had they got worse.”

Quote3

“With some people, it could, yeah. It could affect their frame of mind and their negativity, so that could have a derogatory effect on them possibly, yeah, to realize that they’re going downhill as opposed to uphill. Some people might need that to make them realise that they’re getting worse because some people are in denial over their illness and don’t accept it even though they might see it. And to have it in black and white on a bit of, on a tablet, might be what they need to give them a bit of a kick start…”

T2P02_Service User

“…Once I got used to the sequence of what buttons I needed to press, then I liked the facility to record numbers against various criteria week after week, and the fact that it was possible to compare responses across weeks.”

T2P09_Service User

Quote1

“…after having a session … I’d often think about the low areas and what I’d do and improve them…”

Quote2

” Just for me to be able to identify where the changes are and then try and work out where they’ve come from and so I can sort of see if something’s… in particular beneficial that I may have done in the week prior, or not done the week prior, if it made me feel worse or whatever …just so I can get an idea.”

Quote3

“…I kind of thought it might be a little bit impersonal because obviously it’s on a scale of one to ten kind of thing, it can be a little bit hard to gauge what each value actually means. Yeah, so I was a little bit sceptical at first as to how much information I’d provide … on it.”

T3P01_Service User

”…it highlights what you’re doing really good on and also it highlights what maybe you’re not doing so good on. And the fact that you can physically see from the previous weeks and monitor them, so you can think right, I’ve improved on that, and that makes me feel really good.”

T3P02

Quote3

“So, the 7 was slightly a bit odd, it would almost be more logical maybe to do it 1 to 5 or 1 to 10… it did feel slightly arbitrary and also there wasn’t very much fine tuning on it, it felt a bit blunt at times. So, whether if you did it out of 10 you could have half points as well, because a couple of times I thought, well I suppose it’s about 2.5 and oh, hang on, I’ve got to do 2 or 3.”

T3P04_Service User

Quote1

“So sometimes if you’ve got a spare few minutes and your mind wanders anyway you might think about, I don't know, leisure activities or something whereas you never would have thought about that before, because it’s been brought to the forefront of things … you might think about making a slight improvement on that area.”

Quote2

“… if I did continue to use it would be helpful because you can look back on your previous scores and it gives you like a chart I believe of what you scored in different aspects of your life, like physical health and jobs and stuff. Yeah, I think it’s good to see that if you were a five or six out of seven one week and then one week you’re a two or three then obviously it’s good to look at why and it’s good to have a measure of that.”

T3P06_Service User

“Yeah, that actually made me stop and think how if it was a good thing or maybe disagree … it actually made me stop and think and read it through. I read it through once and I thought, I’ll read it through again just to try and get my head around what the question was asking. Yeah, it sort of made me think about it.”

THEME 4: EMPOWERMENT AND POWERLESSNESS

T1P07_Service User

“And it also makes you know what you are expected to do. This is what I said... This will help me to know that no-one is forcing me to do anything but this is what is expected of me to do… So I know we are all working into one goal.“

T1P10_Service User

“It gave me an opportunity to look at areas of my life that I could control myself and, but there were some areas that I needed help with… so that was different because before you just put all your eggs in one basket first and this way it was all itemised really and so, OK, and this is, how do I get out of this hole…”

T2P05_Service User

"… say I wasn't happy with my medication and if you put one and saying why this rating and not a lower one, well I couldn't put anything less than one. What is working? Nothing, because I'm really not happy with it. Looking forward, best case scenario, change my medication. What can the clinician do? Nothing, because they don't want to change my medication right now. So … running through it like that is really pointless and a bit disheartening, it's like, yeah this is still a problem, yes, still nothing can be changed right now. And even though, you know, I could agree with that outcome it's still disheartening running through it and seeing I'm still not getting anywhere."

T2P09_service user

" … I think the most positive thing was agreeing on the actions, so that there was a specific thing to do, which could be done and was done**."**

T3P01_Service User

"…it is good because … I didn't realise how many of these things were affected, and it's good also for me to set goals and see what I've improved on. Because you forget, you do forget how good or bad things are, so from that point of view it was good to see through the weeks, what I had improved on. It gives you a bit more confidence to think you're going in the right step as well."

T3P09_Service User

":…she might say, OK, well, your job situation's always scored low and she might dwell on that but again it's difficult because my job situation doesn't, there's not a lot you can say. When I'm not allowed to work and I want to work what's the alternative?"

THEME 5: THE IMPACT OF TECHNOLOGY

T1P07_Service User

Quote1

"I love computers anyway so it's easier. It's just one click and everything has gone so I will not say I don't like anything about it. I do love it actually…I know that my information is saved there and … unless maybe someone rob … this thing but I know it's safe and secure there."

Quote2

“It helped him to spend more time with me. Not unlike before when he comes he has to write and spend time on writing, and he will ask me to repeat … when I talk he has to take time to write but with this we’re both doing it together and whilst he’s talking I’m also talking and he’s putting it in the system so this is more easier than when he’s writing.”

T1P08_Service User

Quote1

“…now she’s got the tablet, she (*name of clinician*) does more stuff because she remembers. It’s in the tablet so it’s harder for her to forget.”

Quote2

” I think, also , on (*name of clinician*)’s part needed a little bit more training on how to use it because she struggled a little bit on using it. So it kind of… made us last a little bit longer on using it because she didn’t understand how to use it properly. Once she started to get a little bit of a handle over it the session was almost finished.”

T1P15_Service User

“I suppose interaction went because you’re just sitting looking at a, what do you call it?…tablet…and touching squares or whatever to answer the questions, I suppose the social interaction went in that respect. So probably that was on the negative side…Not a lot, a little bit … she would sort of be looking at something she might have to do or whatever, as opposed to interacting with me. But then if I was concentrating on using that, I probably wouldn’t be hearing her properly anyway, because I was thinking about something, using the tablet and answering the questions.”

T2P05_Service User

“… there was lots of pressing of buttons and to and from trying all, this is the wrong screen, is it this one? Is it that one? Oh, it's all gone. Oh, it's back. It was lots of that, so it, I don't think, especially my CPN who was trying to lead with it, I don't think she found it user friendly.”

T2P06_Service User

“I found when I was answering the questions it took a while for it to register. And then when you were stuck, when you read a question and it says yes or no and we put tick to go on to further information it wouldn’t take us any further. We tried that on two or three occasions on the same particular question. It was one of those questions and it just didn’t want to take us any further. And that’s as far as we got on, yeah, on every occasion.”

T3P01_Service User

Quote1

"I think, like I said before, the main thing is monitoring your progress and … you can also forget how good things were, how bad things were from the previous session. Obviously, care co-ordinator takes notes and things but obviously you've physically got it in front of you. So it's a case of just getting the tablet out and showing you and you can physically see it. Rather than her trying to verbally explain it which sometimes can be a bit more difficult."

T3P02_Service User

"…I had CBT a couple of years ago, and there was a lot of form filling for that and box ticking, and it felt quite like that. Because, I suppose, I'd always assumed, the forms, they disappeared in somebody's file never to be seen again. So, actually having it on a tablet where you could at least go back and compare and actually track whether, that's better or oh damn, there was a bit of a slide back on that…"

T3P04_Service User

Quote1

"…I do feel like we would have talked about that stuff anyway if I'd have been honest to bring it out. But I think putting it down on an app or something like that is easier because sometimes it's easier to do that than to verbalise it. But if I was good at communicating how I was feeling then I wouldn't necessarily have needed the app….I think there are probably some people that would find it easier to put it on an app than to actually say it out loud. So it could well be different for other people…"

Quote2

"I didn't use it often but I think it would be really beneficial for people that, in the future that use it. I think a lot of things nowadays are easy to keep track of so when it's using technology you've got all your records there so you're not having to have loads of paperwork so I think that would be good."
