# Supplementary material for: Acceptability of a technology-supported and solution-focused intervention (DIALOG+) for chronic depression: views of service users and clinicians
Source: BMC Psychiatry. 2021 May 20;21:263. doi: 10.1186/s12888-021-03256-5 (PMC8139109; doi:10.1186/s12888-021-03256-5)
Supplement: Supplementary file 5 — Additional file 5. Mental health professionals quotes: Quotes from the interviews with mental health professionals. [file 12888_2021_3256_MOESM5_ESM.pdf]

SUPPLEMENTARY MATERIAL: ADDITIONAL FILE 5

MENTAL HEALTH PROFESSIONALS' QUOTES

THEME 1: DIALOG+ STRUCTURE

T1C08_clinician

"A classic care co-ordination session, the client comes to see you to discuss whatever problem he has or whatever issues or needs he has. There is no framework defined out there, whereas with the DIALOG+ intervention, number one we open discussing the action which was decided and discussed in the previous session, the previous week or previous two weeks. So this is number one point of focus. Number two, the second point of focus, now we have here a point of discussion about what we are going to do now and what we are planning to do by the end of the session. Well, this is amazing."

T1C09_clinician

Quote1

"…it becomes more focused, isn't it… if you wouldn't use this, and you just have a very free session where you just let them come with whatever, it can feel very much like overwhelming sometimes and there's so many different things that you almost find it hard, well, what shall we focus on? So I think this is definitely helping … just making a bit more I guess targeted and it's easier to actually pinpoint what steps you can take to make things a little bit better..."

Quote2

"I think it wasn't difficult to use. I think it's just one of the things that you need to get into the habit of using and I think also for clients who's not used to that way of working it takes them a bit of time maybe also to just get used to that idea and that structure because it's … I guess you kind of steer the focus a bit with the solution focused, with those four questions and I think that's just something that might feel a bit unusual for the client if they're used to just come in and just burst out everything that's been going on in their lives and that's normally what happens straightaway."

T2C01_clinician

"… I think this would be great if you were using it when you started off working with somebody from new…I think this would be really helpful because you could then get that structure in your head from the

word go and it is quite a helpful structure actually…you'd just find your own way of getting through that, keeping to the structure, but your own style would then become a bit more natural."

T2C02_clinician

Quote1

"So I think it works better, in my experience, with somebody completely new to me than somebody I've been working with a year when really I know and they know that I know a lot of their situation. So it was good to be completely green and innocent and go, oh what do you do for work, or stuff … I found that easier really and more engaging."

Quote2

"I found it made the sessions very long .... I'm trying to keep it succinct to an hour yet when we were using this, although very useful and it flagged up some things that were of use for me to know, it extended it by about an hour so it wasn't quick."

T2C03_clinician

Quote1

" It did feel quite weird because it's something that I wouldn't have naturally done, but I think for this person in particular it was needed … I came away from the session thinking that was actually a really good thing for her because I could sit there for an hour and a half and not get anywhere so it was quite nice to have a focus."

Quote2

"I think it might be easier with patients that I didn't know because they wouldn't be used to my way of working so it wouldn't be a shock to them that I'd introduced this app…"

T2C06_clinician

Quote1

"…it was good and it felt that it… it just keeps it at… those are the questions, that's what you were to answer and it kept you focused….

Quote2

"Yeah, I think it's very good. I think it's something you do together and it keeps you structured so you don't go off into different directions and you can talk quite openly about the cases and it covers everything anyway."

Quote3

" I think it's a really good idea. I think it helps. I think it improves having a structure and not going off at a tangent so it keeps you on that road."

T3C01_clinician

Quote 1

"…it's a really nice way of breaking those areas down so it doesn't feel as overwhelming as it might… So it might be that somebody's got something quite good going on in one area in which case maybe that is something you can focus on as a positive or as strength whereas other areas might not be so good. There might be something in their life where actually things are going quite well and that sometimes we don't always focus on".

Quote 2

"… in actual fact the clients that I used it with were well established clients and I think that in itself could potentially be an issue.... But I think had I not been and were they quite new clients I think it would have been quite helpful in starting to form that collaborative relationship because it is something that you focus on together and talk about together."

Quote 3

"I think sometimes it was a matter of time really because given that, as I say, with the two clients I was working with we already had a therapy structure set up… sometimes to come to the tablet and the app felt like we'd already perhaps had discussions around the things we were then looking at within the app."

T3C02_clinician

Quote 1

"There are some pluses and some minuses. I actually find the format on the device quite limiting, but that might just be a personal way that I work. For example a paper format would actually be more useful because you could scattergun it and write down stuff as you go along but I guess that's kind of defeating the whole point of it being so structured and helpful… sometimes people find the really structured approach stifling … it's a thing to get someone who's already a bit thought disordered sometimes, yeah, a bit stuck on the same bit. But, yeah, there are some good bits to it. "

T3C03_clinician

"I think maybe it was a little bit more structured and it helped structure and break down the areas. And also, maybe for the patients as well to think about the different components in their life that are really important for their functionality and the overall hopefulness and enjoyment and the importance of that for recovery…. "

T3C04_clinician

Quote1

"I thought it would be a real good way to structure visits with clients and obviously the way you converse with clients…I thought it would be really handy to have that structure…I thought it would give me more oomph to run sessions with."

Quote2

"I work on a totally different unstructured approach … and that, for me, it was too structured. Like you say it was quite good to have those pointers but, no, I don't work like that."

T3C05_clinician

Quote 1

"... And because you're using an app you don't get lost in the fluff of the conversation sometimes."

Quote 2

" …having to use an app and having to use anything like a manualised intervention you're always constrained in terms of what you're talking about and focusing on, but it's liberating in the fact that it is allowing me to really analyse things and go into lots of detail. So as soon as you get past that bottleneck of I'm using an app it actually opens up all these aspects of their life where you can go, well, actually people really struggle with their housing but they don't really talk about it but actually there's a lot of quick wins we can make here. So that's quite liberating because it felt like an easy way to get to the core problem quite quickly but you just had to go through the app if that makes sense. So I'd say overall it was quite a liberating kind of experience I thought."

THEME 2: THERAPEUTIC COMMUNICATION

T1C07_clinician

"If anything, to me it kind of, it enhanced the working relationship in that…because the questions are general for everyone the person doesn't feel so much like it's a personal thing about them. So on that score to me it's a good thing."

T1C12_clinician

"…it got on way with the usual work as anything else, anything new. Sometimes we feel a bit uncomfortable but because it's helped me with how I work with my patients and also brought in positive results, with patients challenging and reflecting on things that we wouldn't usually look at, so it helped in that way."

T2C01_clinician

" …I think once we got going it did, particularly with the first man, it did actually open up a couple of areas for discussion that I hadn't thought of and that was good. He'd done that which I thought was very helpful."

T2C04_clinician

Quote1

"In some ways it's useful because it may open up a conversation that you might not have had. That doesn't happen very often, but it did happen on one occasion. "

Quote2

" So, I just think it's an expensive way of doing what we're doing, and it's quite time consuming really. Because you can get a lot more done if you're just chatting with people...than formally putting the pressure on them to try and come up with an answer which was a struggle for one of my ladies."

T2C05_clinician

"Encouraging the clinician and the client to take a broad view of their situation, especially sometimes when it's a long term client, you can get into a bit of a rut of just checking on mood and so forth and this takes you a lot wider than that."

T3C01_clinician

"…if that's a conversation that perhaps you feel quite trepidations about having with a client or you think that that might be quite a difficult conversation, being able to focus on each of these domains in succession and then coming to that …however they might rate that, once you explained what that means can then facilitate a discussion, around risk for instance. So having something to rate and then facilitate that discussion I think can be quite helpful."

T3C02_clinician

Quote1

"And actually someone I'm working with right now, if you try and put everything into different components it's too hard for them to do, and they've got major depression. I think you could end up wasting a lot of time going round and round in circles because you're prompting them on a topic that then brings up the same kind of answer…."

Quote2

"For some people who don't like to talk a lot, and I can think of someone who this would have been good for… it was a bit like pulling teeth, it would actually be a really good, right, let's have a go at this one."

T3C03_clinician

"I didn't feel constrained and maybe that's because sometimes I felt that it maybe led on to something else that the patient wanted to talk about so I would incorporate that. Or it might lead on to a question about some other part of their treatment. But I think it's for that, for me I would go with what, be led by the client and then be able to come back to it."

T3C04_clinician

"With the one that was a bit more engaged with it, she didn't like it. …She felt it was too structured then. She felt it was better just to sit there and talk like we normally would. So I wouldn't say it affected our therapeutic relationship but, yeah, in that way I suppose it did a little bit. She just felt it easier… just to sit and chat like we normally would…"

T3C05_clinician

" … for me it's slightly jarring after knowing the person for a couple of appointments and then going, oh, we're going to try this app now …I was building narratives with them and then suddenly it was like stop, we're going to use this."

THEME 3: REFLECTING AND MONITORING

T1C08_clinician

"…after they did that comparing with the previous scores automatically created the narrative in front of him because he's looking and saying, oh, yes, oh, it's improved... What contributed to that? What happened? And the client, oh, did this. It's, oh, look at this. Because of the scoring and the comparisons… which is there in front of the client automatically my work is done and I don't need to do anything any more because the client says… OK, yes… there are some assets…there is some capital that I accrued since our last session, this is great. So, yeah, it's changed. The comparison can shift the practice through another level."

T1C09_clinician

"It is so easy for clients to get into a problem focused way of talking, and they've got issue with this and this and this. So when you steer them more towards thinking about these kind of questions like what's working it's just a different way of thinking. But actually it makes them think a bit more and actually it's easier in a way for them to identify things that could improve things for them.

T1C12_clinician

"It takes a bit of time but I think it's more useful…because you're having prompts of all these different questions. Some of them we actually ask them but rating themselves as well helps more. Because we might ask the question, if they say yes or no, but when they rate themselves and also when you compare these ratings it really helps to see where they're actually at."

T2C01_clinician

Quote1

"I think it's a great way of helping people whose whole mental processes are very slow to be able to take the time to think about and do those things … "

T2C03_clinician

"It might help gather information quicker. Yeah, I think some of the questions like how satisfied are you with your physical health or your accommodation or your job situation they're quite specific and with numbers as well you get a good indicator".

T2C06_clinician

"Yeah, it was nice to see that you could see … how the changes had happened or how they'd commenced or where one was a little bit better at the beginning and then was a little bit worse at the end and why was that. But you were putting it into, because it was over a three week period some weeks things were good and some weeks things weren't so good. So it wasn't a catastrophe if you see what I mean."

T3C01_clinician

Quote1

"And I think it's quite a helpful way to get people thinking about their difficulties, this idea of you've rated that five or whatever. What would need to change to make that six and just having somebody just think about that because often people don't really consider the reasons why they rated something. It's, oh, I'll just stick that down as whatever."

Quote2

"One of my clients was saying, well, you can't do a half. And I said, no, it's a shame, isn't it? Because he might have put it up by half but he couldn't so he left it as it was. So it doesn't always capture little tiny increments of change that might be happening."

T3C02_clinician

Quote1

”People found it hard to nail down what the question was wanting. The one on lifestyle, what was it, some of the questions were a lot more difficult to just hone in under that heading. It wasn’t quite as simple as putting a number on it if you know what I mean.”

T3C03_clinician

Quote1

“…I think breaking it down into smaller, kind of smart goals, I think is really helpful for the clients. And I think to see the scale and if there’s an improvement I think that’s good to quantify that.”

Quote2

“You can look back and look forward and really talk to them about what is it that’s different now and what have you done to achieve that…I did find that helpful and the patient found that helpful as well, I think, from what they said to me.”

T3C04_clinician

Quote1

“… I think it’s difficult for some people to put some of those things on a scale then and certainly with the couple that I did it with the scales were often the same. Each time I did the session it was the same. There was not, obviously not much change.”

Quote2

“I think just in general, yeah, found it difficult to relate really. Maybe that’s just about it being a new way of them viewing their illness and viewing these things really and trying to put it on a scale, maybe, may have just been difficult for them.”

T3C05_clinician

Quote1

“… that was really useful because it allowed us to really drill down into the specifics of it. … I suppose it took a bit of a pressure off me to kind of translate what they were saying into actual meaningful clinical stuff because people say, oh, my sleep is rubbish and they’re actually getting OK sleep. And they’ll say, yeah, my appetite’s fine, I eat my rice cracker every lunch, and you think, oh, hold on. So you can really explore things in a lot more detail and take the time to see it and then when they’re quantifying it you can go, well, there’s a big discrepancy there.”

Quote2

“I suppose my style is very conversational and interpersonal and this gave us a common focus between the two of us to focus on objective changes and things like that as opposed to it being a case of me trying

to pick up from a conversation how they felt their sleep was improving or their relationships and things. So it felt the therapeutic relationship became a bit more scientific but maybe in a good way."

THEME 4: EMPOWERMENT AND POWERLESSNESS

T1C07_clinician

Quote1

"…the patient could see that you're trusting them with the tablet to input it themselves. They could see that as a positive thing because some of them obviously if they're scared of computers or not computer literate. But for some I can see it as an empowering thing as well. It's like almost giving them ownership of their own care and about how they are and where they want to go, what they're doing. I think it's a very positive thing."

T1C08_clinician

Quote1

"Now I'm not the powerful person in front of you, my client, who have the answer and I have the ways to organise… this is the most important fact that developers have is bringing to the sessions a symbolic element demonstrating to the client, look, this is your plan. Dependency is not inside me, the co-ordinator. No, it's you, it's in front of you. What you want should be there and this, making it concrete, it is a very powerful way of narrating this to the client."

Quote2

"Having a client in a material way collaborating with their own co-ordination of care … this is amazing. This is a dream come true in this sense. You're giving the client ownership again over their plans, giving the client capacity to create a plan out there in a way that he can transfer to other places."

T1C09_clinician

"… it makes the client a bit more motivated to … follow through the action plan for the next meeting and they … almost take a bit more responsibility for their own recovery. So that's what I found in particular with (*service user*) that she was almost … competing against herself. … if we had an action plan about her incorporating more exercise in the week and we had a target, say, that she would do it twice or three times per week, and if she had done it more that was a great sense of achievement."

T1C12_clinician

"I think it's good, and also because it gives the patient a bit of independence that they're doing it on their own… "

T2C01_clinician

Quote1

" …seeing on the second time that things were actually worse than the first time was quite difficult for her because I think she felt this is showing me things are going backwards even though maybe they weren't necessarily going backwards when you delved into the things but her immediate response to it was that they were going backwards. But I think when you unpacked it you could have argued otherwise."

Quote2

"The leisure activities she also was a bit unhappy about it because she felt that having it there and having to rate it that there was some expectation that she would be having to participate in a number of leisure activities, and when she wasn't, she worried that she would in some way be judged because of that."

T3C01_clinician

Quote1

"…it very much involves the client putting down their own ratings for instance and you can hand over the tablet to them and they can take that control of something …"

Quote2

"…there's quite an emphasis on how can we get you changing, how can we get you moving forward, how do we get you more? And that's great but I can imagine how if it's not done in a very sensitive way it could be quite invalidating for someone with depression where often what you see is a withdrawal from activity and isolation of themselves, and not wanting to engage for all sorts of reasons."

Quote3

"…I think probably particularly in secondary mental health the problem is so deep seated and chronic that shifts aren't going to happen too quickly and the person's perceptions of their difficulties is sometimes understandably far worse than perhaps as a clinician. You can see changes in that person but they're maybe reporting things and feeling quite wary about saying anything's better perhaps"

T3C04_clinician

Quote1

"Well, exploring options obviously is always difficult because they don't see that there's an option. They don't see change in the things that they describe so obviously sometimes exploring those options, exploring the options can be quite difficult for them. But, no, the four steps are very helpful actually …"

Quote2

"…. there was a couple of questions in there, like, the job situation was a no go with the two clients that I chose then. Obviously that may be more relevant to other clients but the two that I chose… the job situation and the family… they're always a bit… become sort of a bit stuck …"

T3C05_clinician

Quote1

"As an approach it was breaking it down for the patients especially … I think they got the point of it, this isn't a case of just tell me your problem and I'll give you an answer. It was actually, look, what are the barriers to you actually getting to this change? So I think they could see that, the identifying of potential problems and pitfalls…"

Quote2

"…because symptomatically of depression it's very difficult to look at best case scenarios because everything seems pretty lousy. So sometimes the best case scenario is, that's like, oh man, you're really underselling it here…"

THEME 5: THE IMPACT OF TECHNOLOGY

T1C07_clinician

"…they can go back to it because the information is there, it's saved… The person can feel that they're being listened to and also the information they've given you is there and it's concise the way they want it to be. I think on that score it's good."

T1P08_clinician

"I can see issues with the technology itself. With the app itself it can be improved some bits and pieces on there, actually, become more straightforward as you expect when you think about an app. But (*to*) have the technology as the interface, as a mediator, it was not a barrier at all, it's the opposite. It gave focus."

T1C09_clinician

Quote1

"…so with clients that I've already started working in a different way and then introducing the tablet, I don't know, it maybe feels a bit sort of…. I found that with one other person that I did it with to communicate via the tablet can maybe sometimes lose a bit of that relationship or communication. I don't know. That is because you do it via the tablet."

Quote2

"So it takes a bit of time …if they were about to say something you kind of have to stop them, hold on, I just need to see where we're at on the tablet so that I can either note down an action point or … if it locks itself where you have to then retype the password…. But those things are the things that I've had and then you're kind of sitting there trying to work out where you were and navigate yourself. If you're not

used to using it all the time and then maybe you lose a little bit of … the client might be a bit put off with that, that it takes a bit of time or that it's not the right flow…"

T1C12_clinician

Quote1

"And doing it on the app is better than paper file because you're just carrying one thing instead of carrying a lot of papers with you…"

Quote2

" When it was working it was good I could manage to navigate, but when it stopped working that was a bit of a struggle and when that happens, usually even with the computer …I'm wary about. Some people are very exploratory they touch that, touch that, then find a solution. But with me, I just sort of stop dead, went back to using the paper."

T2C01_clinician

"…one of the things that I didn't like and I don't know how you'd get over it is the proximity with which you, how close you had to sit to the patient when you were doing it. That felt quite uncomfortable. We were right eye ball to eye ball really because, again, you have to sit close to somebody to help them to do it, especially when they're new to it. So we were having to sit very close together and go through this quite slowly, almost eye ball to eye ball, and I found that quite uncomfortable. … And it's just in any conversation there's just naturally a certain amount of distance… it just kind of works itself out, so that felt a bit unusual for me and I didn't like it."

T2C02_clinician

"…also worried that I wouldn't be able to use the computer, the tablet, effectively because I'm not very good at computers and things…"

T2C03_clinician

"… I think it would have been difficult to use at every single session because it takes away the relationship that you have with this device that's taking over the session…"

T2C04_clinician

"…we have a busy job we have a stressful job. But each time I'd have to make sure that it was charged up well enough and that it was working properly. So it was another extra time just making sure that it was going to do what I needed it to do without any hiccups in the session with the patient."

T2C05_clinician

"…I'm not the most organised person in always having the charger and the lead, knowing where it is, having it ready. In fact I did always have it ready but it wasn't all that easy because it's yet another device because I've got a laptop, a mobile phone, the Trust iPad, notebooks and then you add as well this extra tablet, it's too much really for ideal purposes."

T3C01_clinician

Quote1

"…one thing I did like about this piece of technology was the fact that you can go paperless with it because again thinking about the reams of paperwork you can get through doing psychometric measures with people which is lots and lots of paperwork and lots and lots of trees."

Quote2

"I think the barriers for me, and this might just be me not twigging in terms of how to use it technically, was the fact that the screen would lock quite quickly and I didn't manage to figure out a way of stopping it doing that."

T3C02_clinician

Quote1

"So particularly with participant 1, she's got low confidence and can't always see the improvements she's made. So in showing her that she's actually gone up a number … over time, you'd have to do it. So from when I first started with her she'd have been right down the bottom for everything and actually pointing out that she's a lot further down the line is actually quite useful. Nothing like some facts… sometimes having it there in front of you is quite useful."

Quote2

"Yeah, it doesn't fit in with my personal style of nursing really but (*for*) other people it would. I like the principles behind it. Yeah, it was the technology bit that was constraining. It was the having to sit and input stuff and you're losing that contact time and chance to observe someone."

T3C03_clinician

Quote1

"So with the first patient I think I struggled to use it and I just couldn't get it to go back and forward and I think that's because there was a gap in when we did the training."

Quote2

"…it wasn't helpful that I couldn't negotiate the tablet very well because we couldn't do it properly I think … to people like me that aren't very good with technical things it might have been good to have maybe had some role-play during the training. I think that might have helped me more."

T3C05_clinician

Quote1

"…one of the patients in particular … was quite explicit to say … I can use computers at home and, well, actually I'm here to speak to you as a human being and not use a computer as an app…. So I don't know about my use of the app and the way I integrated into my practice but I think he was just literally referencing like, oh, we're looking at a screen now and the face to face stuff has kind of gone. "

Quote2

"But with the app … there were pauses and just changes and stuff, so it kind of took away some of that spontaneity and rapport…. so it changed (DIALOG+) in the way the conversation happened slightly but it wasn't one of those things I walked away going, oh my God, what a disservice, it was just more like the reflection, supervision stuff you bring up as a practitioner rather than genuine clinical problems I suppose."

Quote3

"And then the other lady she used an iPad religiously but she struggled with, I think it was just the sliding scale kind of thing. It was maybe not sensitive enough in terms of the touchscreen or over sensitive or something but, yeah, she struggled a little bit just getting the fine tuning because it got to a point where I think, well, some of the stuff she said, oh, I think that's a six and we put a seven and then she'd go, oh, sod it, I'm going to leave it in there."
